# Topic modeling and clustering in the trace data-driven analysis of job demands among teachers

**DOI:** 10.1038/s41598-023-45356-0

**Published:** 2023-10-21

**Authors:** Tiina Kalliomäki-Levanto, Ilkka Kivimäki, Pekka Varje, Olli Haavisto

**Affiliations:** Finnish Institute of Occupational Health (Työterveyslaitos), P.O. Box 40, FI-00032 Helsinki, Finland

**Keywords:** Psychology, Environmental social sciences, Mathematics and computing

## Abstract

Psychosocial work environment characteristics like job demands have traditionally been studied using survey data. We propose an alternative approach utilizing work related trace data collected from the information systems that employees use to achieve organizational goals. We analyze the job demands of teachers from two universities of applied sciences using trace data collected from the educational online platform Moodle over a period of 90 weeks. The data contain pairs of targets and actions (like message_sent) performed by teachers on Moodle. The timestamps of the target-action pairs allow us to study the dynamic nature of job demands, which is not possible by using periodically collected survey data. We show how trace data can be used to analyze processes related to job demands using data-driven approaches. We have identified topics, themes, temporal processes, and employee clusters from Moodle data representing the work tasks of teachers. The information obtained is action-oriented, context-specific, and dynamic, meeting the current needs for information about changing working life. The approach we have provided could be widely utilized in organizations as well as in research on occupational wellbeing. It is useful in identifying targets for intervention and it could be expanded to include prediction models on different outcomes.

## Introduction

Karasek’s^1^ demand-control model predicts that mental strain results from the interaction of job demands and job decision latitude (control). According to the model, the combination of low decision latitude and heavy job demands are associated with mental strain. Therefore, the reduction of mental strain can be achieved by redesigning work processes to allow increase in control without reducing the job demands such as workload, which are associated with organizational output levels. Following Karasek’s proposal, the numerous intervention studies that have tested the model since 1979 have focused mainly on the control-dimension of the model and not on the demands.

However, in a recent contribution Elovainio et al.^2^ suggested on shifting the focus on the demand-dimension as a target of intervention. In their analysis of the psychosocial work environment, they identified job demands as one of the core factors which could constitute a valuable target for workplace interventions. Improving central “upstream” factors such as job demands could result in a spread of positive influence also in other parts of the psychosocial network. More evidence on the dynamics of job demands could allow informed decisions about whom, when, and how to intervene to improve work environments and occupational wellbeing^2^.

When constructing the scale of job demands, Karasek^1^ defined job demands as follows: “The goal in constructing the scale of job demands is to measure the psychological stressors involved in accomplishing the workload, stressors related to unexpected tasks, and stressors of job-related personal conflict.” (p. 291) In previous research utilizing Karasek’s model, the demand-dimension has been analyzed mostly by relying on surveys based on employees’ own subjective perceptions of their work^3^. These surveys have been based on following features^1^: work fast, work very hard, lots of work, not enough time, excessive work, no time to finish and conflicting demands (p. 289).

The validity of perceived information about psychosocial factors such as demands and control collected through questionnaires has been evaluated in exposure studies. According to Solovieva et al.^4^, the validity of the perceived information about job demands was weak. They argued that there is a need for more detailed information in job demands, but most studies still utilize perceived because more detailed information is not available.

The need for more objective information about job demands, such as job analyses or organizational records, has been pointed out in several contributions^3,5,6^. In a rare example, Rau et al.^6^ used objective information about job demands when exploring association between job demands and depression. The information about job demands was based on Task Diagnosis Survey^7^. According to Rau et al.^6^, this method could be considered “objective” because “job analysis experts rate work characteristics independently of the employee’s perception of his/her work task” (p. 93). They were able to show that the objectively defined demands were significantly associated with depression.

Spector and Pindek^3^ have pointed that digital big data could constitute a new source of information about work and work environment in organizations. Data-driven approaches utilizing big data sources could have the potential to yield valuable insights into many organizational phenomena. These approaches could be used to recognize patterns from data and predict important outcomes such as illness or injury^3^.

In addition to the need for more objective information about job demands, there is also a need for dynamic and process-like information that could capture the characteristics of contemporary work^3,8,9^. To observe processes, one requires temporal sequences that must contain a beginning, an end, and intermediate steps^8^. Although survey information is often longitudinal, it is also static, gathered at certain time points. Using survey data, it is not possible to discover the process of work in the way enabled by timestamped trace data. The information in trace data has a timestamp, making it possible to discover the temporal progression of activity as processes^10^.

In contemporary work life, accomplishing the workload increasingly occurs with the aid of information systems (IS). The use of IS leaves traces in the log data of the systems and these traces can be understood as information about the job demands. With trace data it is possible to define an event containing four features: timestamp, actor, target and action^11^. An example of the target and action pair found in the trace data could be “message_sent”.

The use of trace data in the analysis temporal patterns has been approached from a pragmatic, action-oriented framework that focuses on how patterns emerge from agentic action, thus avoiding overtly structural and abstract theorizing^12^. Digital trace data has also been approached from the perspective of critical realism that recognizes trace data as a by-product of monitored activities that can be used to empirically observe actual events generated by real mechanisms in a specific contextual environment^13^. Following these perspectives and combined with powerful computational methods, digital trace data can be seen as an important indicator of work demands. With trace data it is possible to get action-oriented and context-specific information about the practical content of work activities and their temporal progression over time. Instead of subjective statements about heavy workload and a lack of time, trace data reveals objective information about peak weeks and deadlines.

In this article, we analyze job demands in more detail taking into account their dynamic nature. For this purpose, we use trace data stored in information systems that employees use to achieve organizational goals. We analyze the work demands of teachers from two universities of applied sciences (Organization A and B) using trace data collected from the educational online platform Moodle^14^ over a period of 90 weeks. The data contains pairs of targets and actions performed by teachers on Moodle. The timestamps of the target-action pairs are also included. The data can be considered to reflect the nature and amount of the work activity of the teachers.

The analysis of work demands is achieved through a twostep procedure. First, using topic modeling, we identify topics from the teachers’ work activities and combine the topics into interpretable themes. The activities of each teacher are used to construct the distribution of themes for each week and the weekly distribution of the themes for each teacher forms a sequence that can be analyzed as a process.

Second, we define a dissimilarity measure between teachers based on their processes. The dissimilarities are then used to cluster the teachers considering the period of spring in years 2019 and 2020 from week 1 to 22 before the summer holiday. For interpreting the obtained clusters, we visualize the average processes of the teachers in each cluster. The visualized average processes of the different clusters can be named based on the demand-control model^1^. These named processes can be used in the future for finding targets of intervention and for further studies.

## Methods

### Data

Data was gathered from the Moodle systems of two Finnish universities of applied sciences (Organizations A and B) and consisted of the teachers’ activity logs. The data contained 11 and 5.7 million lines of trace data over 90 weeks in 2019–2020 for Organizations A and B, respectively. There were 423 teachers included in Organization A and 320 in Organization B. Each row of the trace data contained the anonymized teacher id, date of the recorded activity, and the performed action and target. Due to the anonymization, the data contained no other information on the teachers (demographic characteristics, specialty fields etc.). Table 1 shows an example of the data from one organization.

**Table 1:** Randomized sample of the Moodle data.

Teacher id	Date	Target > action
< id_54 >	17.6.2019	course_module > viewed
< id_84 >	17.12.2019	course > viewed
< id_255 >	3.6.2020	course_module > updated
< id_284 >	26.8.2020	course > viewed
< id_143 >	18.2.2020	grading_form > viewed
< id_18 >	27.1.2020	course > viewed
< id_273 >	26.8.2019	user_list > viewed
< id_162 >	10.5.2019	user > graded
< id_208 >	9.9.2020	course_module > viewed
< id_303 >	21.3.2019	notification > viewed
< id_247 >	28.8.2020	notification > sent
< id_5 >	16.9.2019	notification > sent
< id_22 >	4.6.2019	user > loggedin
< id_71 >	2.3.2020	course > viewed
< id_79 >	21.11.2019	dashboard > viewed
< id_247 >	15.4.2020	dashboard > viewed

As a preprocessing step, a set of common target-action pairs were removed from the data since they corresponded to general use of the system instead of a specific task performed by the teacher. The removed pairs were: user_loggedin, user_loggedout, notification_sent, notification_viewed, course_viewed and course_module_viewed. Additionally, weekly activities with less than 25 recorded target-action pairs for a teacher were omitted from the further analysis.

### Clustering teachers based on Moodle data

One of our main goals was to see if the teachers in the data could be clustered in a meaningful fashion and to discover possible interesting behavioral differences between the clusters. We approached this problem in two phases. First, we performed Latent Dirichlet Allocation (LDA)^15–17^ to reduce the dimensionality of the data. Then, based on the LDA representation of the data we defined a dissimilarity measure between teachers. The teachers were then clustered based on the matrix of dissimilarities between all pairs of teachers. For the LDA modeling we used all the data gathered over a period of 90 weeks. In the clustering phase we used the weeks 1–22 from 2019 to 2020 that represent the spring semester in universities. We describe this process in more detail below.

As said, the data was first analyzed using LDA which is a generative Bayesian topic modelling method with foundations in text analysis. It defines semantic topics as probability distributions over the vocabulary of a text document collection. A predetermined number of topics are inferred from the document collection so that semantically similar words become grouped into the same topic. The documents, i.e., the data vectors, can then be transformed from a bag-of-words vector representation into probability distributions over the obtained topics. Thus, LDA can be considered as a dimensionality reduction method, where the size of the vocabulary defines the dimensionality of the data space and the number of topics, which is set much lower than the size of the vocabulary, defines the dimensionality of the feature space.

We interpret the Moodle data similarly to bag-of-words text data, where the vocabulary is defined by the set of target-action events that can be performed on Moodle. The collection of events, and their occurrence counts, performed by one teacher on one week is considered as a document. LDA thus produces topics as probability distributions over the set of target-action events, in addition to which each teacher becomes assigned with a topic distribution for each recorded week.

The use of LDA is motivated by two aspects. First, it increases the interpretability of the activity of the teachers, and second, by reducing the dimensionality of the data, it makes computational comparison of teachers with dissimilarity measures more reliable by circumventing the curse of dimensionality.

LDA models were computed with numbers of topics ranging between 10 and 20 and with 50 different random initializations. The quality of each model was assessed with the Bayesian information criterion (BIC). The final model was selected by manual inspection among the models with low BIC scores, and it consisted of 17 topics. The topics of the final model are presented in Table 2.

**Table 2:** Topics generated by Latent Dirichlet Allocation (LDA) for the two organizations.

Topic#	Top target-action pairs for organization A	Top target-action pairs for organization B
1	Dashboard_viewed	Calendar_event_updated course_module_updated
2	Grade_item_updated	Course_bin_item_created course_module_deleted
3	Attempt_reviewed question_manually_graded report_viewed	Grade_deleted
4	Course_module_updated	Dashboard_viewed submission_status_viewed
5	Message_sent	User_list_viewed
6	Course_module_completion_updated	Tag_added
7	Discussion_viewed	Question_deleted question_moved
8	Grade_report_viewed	Grading_form_viewed
9	Grading_form_viewed Submission_graded	Grade_item_updated
10	Course_bin_item_created course_module_deleted	Grading_table_viewed submission_status_viewed
11	Grade_deleted	Discussion_viewed
12	Course_section_created course_section_updated	Submission_graded user_graded
13	User_list_viewed	Attempt_reviewed question_manually_graded report_viewed
14	Chapter_viewed group_member_added user_profile_viewed	Course_module_completion_updated grade_report_viewed
15	Attempt_viewed edit_page_viewed	Attempt_viewed edit_page_viewed
16	Submission_graded user_graded	Webservice_function_called
17	Grading_table_viewed submission_status_viewed	Chapter_updated chapter_viewed

Some of the 17 LDA topics were further combined manually into 5 main themes that were related to different aspects of work involved in teaching and administrating courses in Moodle. The topics that were not suited to any of the five main themes were combined into an extra theme titled "Others". The six resulting themes are defined in Table 3.

**Table 3:** Manually formed themes based on the topics. The rest of the topics formed the theme ‘Others’.

Theme	Topics in the theme (Org A)	Topics in the theme (Org B)
Evaluation	Topic #9: grading_form_viewed submission_graded Topic #17: grading_table_viewed submission_status_viewed	Topic #8: grading_form_viewed Topic #10: grading_table_viewed submission_status_viewed
Courses	Topic #4: course_module_updated Topic #10: course_bin_item_created course_module_deleted Topic #12: course_section_created course_section_updated	Topic #1: calendar_event_updated course_module_updated Topic #2: course_bin_item_created course_module_deleted Topic #14: Course_module_completion_updated grade_report_viewed
Progress tracking	Topic #3: attempt_reviewed question_manually_graded report_viewed Topic #15: attempt_viewed edit_page_viewed	Topic #13: attempt_reviewed question_manually_graded report_viewed Topic #15: attempt_viewed edit_page_viewed
Situation tracking	Topic #1: dashboard_viewed Topic #7: discussion_viewed Topic #13: user_list_viewed	Topic #4: dashboard_viewed submission_status_viewed Topic #5: user_list_viewed Topic #11: discussion_viewed
Final grading	Topic #16: submission_graded user_graded	Topic #12: submission_graded user_graded

The topic distribution representation of the documents, i.e., the teachers' weekly topic vectors, were transformed into theme vectors, i.e., probability distributions over the 6 themes, simply by summing the topic probabilities of the topics in each theme. The theme representations of the teachers' weekly activity were then used to measure the dissimilarity between teachers.

We defined the dissimilarity between two teachers by first computing, for each week in the data, the Jensen-Shannon (JS) distance^18^ between the theme vectors of the two teachers on that week. The JS distance is commonly used for determining the distance between two probability distributions on the same probability space.

The overall dissimilarity between the teachers was then defined by taking a weighted average of the weekly JS distances over the weeks in the data. More precisely, the weights in the weighted average were defined by taking a product of the number of events of the teachers on a given week and normalizing the product with the sum of all such weekly products.

Formally, the distance between two teachers, $i$ and $j$, can be expressed as

1 $$d_{ij} = \mathop \sum \limits_{t = 1}^{T} w_{{ij}}(t) JS(P_{i} \left( t \right)||P_{j} \left( t \right)),$$

where $T$ is the number of weeks in the data, ${P}_{i}(t)$ is the probability distribution over the six themes of teacher $i$ on week $t$ and

$$w_{{ij}}(t) = \frac{{C_{i} \left( t \right)C_{j} \left( t \right)}}{{\mathop \sum \nolimits_{\tau = 1}^{T} C_{i} \left( \tau \right)C_{j} \left( \tau \right)}},$$

where ${C}_{i}(t)$ is the number of target-action events performed by teacher $i$ on week $t$.

The intuition behind the dissimilarity ${d}_{ij}$ defined in Eq. (1) is that teachers should be considered more similar to each other if their theme distributions are similar on most weeks. The weighting based on the event counts, ${C}_{i}(t)$, is motivated by the idea that the similarity of the teachers should be defined more by the weeks when both teachers have a lot of activity.

Note that, although JS distance defines a Euclidean metric between probability distributions, our weighted average dissimilarity is not guaranteed to be Euclidean nor even a metric, hence the use of the term ‘dissimilarity’ instead of ‘distance’, which is more commonly used with metrics.

The teachers were clustered based on the matrix containing the dissimilarities, ${d}_{ij}$, between all teacher-pairs $i-j$. The clusters were obtained with complete-linkage agglomerative clustering^19^. It is a hierarchical clustering method that first defines each data point into its own cluster, and the dissimilarity between two clusters as the dissimilarity between the corresponding data points. It then combines clusters sequentially. First, the two clusters with the smallest dissimilarity are combined into a new cluster. The dissimilarity between the obtained cluster and each outside cluster is defined as the maximum of distances between the outside cluster and the clusters contained in the new cluster.

The agglomeration process can be continued until all data points belong to the same cluster, resulting in a hierarchical cluster structure. The cluster structure can then be studied by focusing on a level of the hierarchy with a fixed number of clusters or a fixed dissimilarity threshold, after which clusters should be considered separate.

### Ethical approval

The research has been carried out in accordance with the EU's General Data Protection Regulation (GDPR), which obliges us in our Institute and also obliges responsible persons in the participating universities of applied sciences. The two universities of applied sciences own the Moodle-data used in our study. They anonymized the data: They gave a random ID to the teacher and destroyed the connection between the random ID and the personally identifiable ID. In addition, only day-level information was taken from the Moodle timestamp to the research data. The Moodle-data contained only Moodle usage-data and no other information. From the Moodle data, we prepared week-level result materials, from which topics were identified using topic modeling. We grouped topics into six themes. Weekly theme distributions were clustered into six clusters. All published results are information aggregated at the population or group level, from which no individual can be identified. The data has been handed over to our Institution by the decision of the controller, and the controller is the university of applied sciences. The authors’ institute and two universities of applied sciences have drawn up the written cooperation agreements. The agreement was signed by persons in management positions in the organizations.

## Results

### The themes of topics in clusters

The trace data was processed using Latent Dirichlet Allocation (LDA) to form 17 topics separately for the two organizations. Table 2 lists the discovered topics and their top target-action pairs.

To combine the two organizations, 11 most important topics were selected and manually grouped into five themes listed in Table 4. For both organizations, the topic with the top target-action pairs ‘submission_graded user_graded’ was considered as a theme on its own because it represents a key part of the teaching work: final grading. Other topics with activity close to zero for most of the weeks and with higher activities on a few, if any, individual weeks were grouped as “Others”.

**Table 4:** Naming trace data-driven clusters as processes of demands from teachers Moodle activity in spring 2019 and 2020. Clusters that contain same kind of information in 2019 and 2020 are in the first three row. Naming is based on information in Figure 2 below.

Clus-ter	Spring 2019	Clus-ter	Spring 2020
1	The portion of the activity in grading-demand (purple + red) increases towards the summer, and the portion of the activity in course-demand (blue) decreases with little portion of progress tracking: ‘Grading increases while course decreases’	1	The portion of the activity in grading-demand (purple + red) increases towards the summer, and the portion of the activity in course-demand (blue) and situation tracking decreases and progress tracking (orange) slightly increases towards the summer: ‘Grading increases with small proportion of progress tracking while course decreases’
2	The portion of the activity in grading-demand (purple + red) increases towards the summer, and the portion of the activity in situation tracking (green) decreases: ‘Grading increases while situation tracking decreases’	3	The portion of the activity in grading-demand (purple + red) covers about a quarter of activity and increases towards the summer, and the portions of the activity in situation tracking (green) and courses (blue) decreases towards the summer with small portion of progress tracking (orange): ‘Grading, as about quarter of activity, increases while others decrease’
3	The portion of the activity in grading-demand (purple + red) remains at the same level from week 4 towards summer and the portion is the largest of the six clusters: ’Mostly grading’	2	The portion of the activity in grading-demand (purple + red) remains nearly at the same level with small portion of progress tracking from week 4 with slight decrease towards summer. The grading portion is the largest of the six clusters: ’Mostly grading with small proportion of progress tracking’
5	The portion of the activity in grading-demand (purple + red) decreases towards week 10 and since then increases towards summer: ’Grading increases toward summer ending to the second largest proportion in week 22’	4	The portion of the activity in grading-demand (purple + red) covers about third of activity and increases slightly towards summer to which progress tracking increases strongly from week 11. The others decrease strongly. ‘Progress tracking increase towards summer’
4	The portion of the activity in grading-demand (purple + red) remains at the same level from week 10 towards summer near half of activity and the other half contain other four activities: ’Half grading and half other activities’	5	The portion of the activity in grading-demand (purple + red) remains nearly at the same level from week 10 and since then progress tracking increases towards summer while activity in others decreases: ’Progress tracking increase towards summer, grading remains the same and other together decrease from week 10 towards the summer’
6	The portion of the activity in grading-demand (purple + red) varies over week ending in a decreasing direction before summer. A similar variation appears in progress tracking activity, which has a clear role in this cluster only: ‘Grading and progress tracking varies together ending up decreasing before summer’	6	The portion of the activity in grading-demand (purple + red) covers about one eighth part of activity with small portion of progress tracking and both stay nearly at the same level over weeks. The activity in spring covers courses, situation tracking and “other” activity: ‘Other activity than grading or progress tracking’

**Figure 1:**
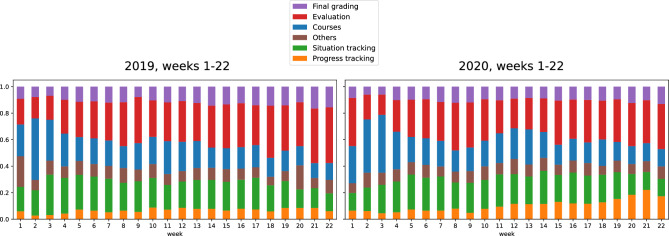
Weekly theme distribution averages of the teachers for spring 2019 and 2020.

For each teacher, the sequence of theme distributions over the 22 weeks defined the process of the teacher. We combined the processes from the teachers of the two organizations into a single dataset that could be targeted for clustering. Figure 1 shows the weekly theme distribution averages of the teachers for spring 2019 and 2020 over the period of 22 weeks. The differences between 2019 and 2020 are relatively small, but the relative share of theme activities changes towards the summer. There is an increase in final grading, evaluation and progress tracking and a decrease in courses, situation tracking and other activities. The increase in progress tracking is more emphasized in 2020 than in 2019.

### Clustering teachers’ processes

**Figure 2:**
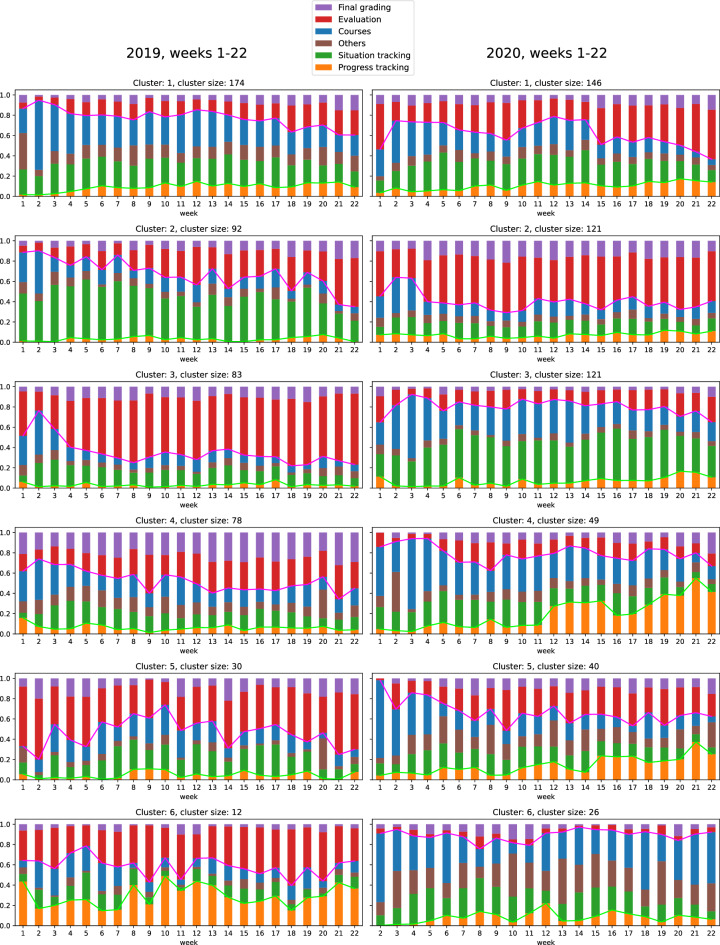
Average weekly theme distributions of the teachers in the six clusters for spring 2019 and 2020. Note that in the year 2020, all teachers in cluster 6 have no data, from week 1, i.e., they performed no or only a few target-actions on that week.

The Fig. 2 shows the average weekly theme distributions of the teachers in each cluster in spring 2019 and 2010. Temporal progressions of activities can be detected as changes in relations between themes over weeks. Week 22 is the last week before summer holiday.

From the themes in clusters, we detect the temporal progressions of activities, which are marked with lines. The themes were also used to name the clusters as shown above in Table 4.

## Discussion

During the analyses we generalized the Moodle activity of the teachers into five themes: ‘Evaluation’, ‘Courses’, ‘Progress tracking’, ‘Situation tracking’ and ‘Final grading’ (Tables 2 and 3). The themes can be divided into two types of activity: one is to view and the other is to do something like updated, deleted, created or graded. To view is dominant in tracking and evaluation while doing is dominant in courses and final grading.

There is a need to interpret the identified themes in terms of job demands and job control. As noted above, the themes ‘Progress tracking’ and ‘Situation tracking’ consisted of activities related to viewing something. We argue that in this context viewing represents job control since it aims at gathering information to support future action such as early intervention to problems in student progression. This kind of proactivity has been defined as a person-level feature^20^ with a positive influence on well-being at work^21^. In the case of the university teachers this tracking represents proactivity enabled by IS.

In the theme ‘Evaluation’ the topics also refer to viewing, but in this case, it is associated with the activity of grading. Therefore, we understand the theme ‘Evaluation’ as a job demand. The theme ‘Courses’ is mainly associated with topics referring to creating, updating and deleting and we understand it as a job demand. The theme ‘Final grading’ contains only one topic, and this topic refers to grading. We understand grading as a key part of the teachers’ work and we interpret this theme as a demand.

From Fig. 1, it can be detected that the relative share of theme activities varies from week to week towards the summer, thus constituting a process with temporal progression over time. The processes appear quite similar in spring 2019 and 2020. Grading demand increases towards the summer and other activities decrease, except for ‘Progress tracking’ which slightly increases in spring 2020.

However, when observing visualizations of the Moodle activity, it should be noted that the relative proportion of the activity in Moodle does not directly indicate the demands of the doing contained in the theme. The “submission_graded user_graded” activity may require major resources from the individual teacher, other persons involved with the activity, and the overall organization outside Moodle. On the other hand, the “course_module_updated” activity may be based on an already existing course and the update is a routine task that does not require so many resources outside the system. Some target-action pairs can mean only one click, while others require hours of work. Therefore, a slight increase in activity around one topic, even if the relative share of the topic is small compared to other activities, can nevertheless indicate a significant increase in demands.

An important characteristic of work in the educational sector is the need to complete all activities before the summer holiday, which may be associated with enhanced time pressures a few weeks before summer. In the next part, we analyze the processes in more detail and observe what kind of peak weeks emerge before summer.

One aspect of process thinking is the ability to name the process and we implement it in the following way. We compare the clusters shown in Fig. 2 and interpret the changes in the relations between themes over weeks as temporal progressions of activities^10^. In Fig. 2, we draw attention to two sets of themes. The first one is ‘Evaluation’ and ‘Final grading’ that represent the grading demand. The other one is ‘Progress tracking’ that represents proactivity (Table 4).

To extract detailed information for intervention, we continue to interpret clusters from the perspective of content and change in processes. The content of “mostly grading”- clusters (2019:3, 2020:2) may correspond to “lots of work” in Karasek’s model^1^ and thus represent high job demands. ‘Grading increasing towards summer’ appear in 2019 in clusters 1 and 2, and in 2020 in clusters 1 and 3. A peak week represents the climax of the increasing trend after which the activity decreases. In 2019 grading demand has peak weeks in cluster 2 (weeks 14, 18), cluster 4 (9,21), cluster 5 (2,14,19) and cluster 6 (9,11,18). In 2020 grading demand has peak weeks in cluster 3 (5), cluster 4 (8, 20), cluster 5 (2,9,17,19) and cluster 6 (8). The peaks related to grading activity (evaluation and final grading) may be seen as relevant targets for intervention.

As noted above, we interpret progress tracking as proactivity^20^ that may be associated with enhanced well-being^21^. Parker et al.^20^ defined proactivity as a person level feature, but progress tracking is also a system-level feature, being a part of the teachers’ Moodle activity. If Moodle enables teachers to prepare for future activity such as helping students in difficult situations, the target for intervention should be the design of the system so that even better proactivity becomes possible. Process tracking has a bigger role in 2020 than in 2019. In 2020 activity in progress tracking increases in all clusters after week 10, which was the beginning of the COVID 19 pandemic. From that moment on work in all sectors as well as in education changed to virtual and digital platforms as far as possible. When teaching became remote and face-to-face interaction as a source of information on the student progress disappeared, the ability to do something similar in Moodle became more relevant. The findings suggest that the pandemic transformed teachers’ work especially in relation to progress tracking.

However, the relation of progress tracking and grading with proactivity and job demands with IS needs further research. Progress tracking may also contain elements of job demands while using Moodle for grading may include elements that enhance job control. Further research is also needed to strengthen the ability to identify relevant targets for interventions. By attaching health-related or other outcome data to the Moodle data it may become possible to analyze how do the clusters differ, for example, in terms of sickness absences, employee turnover, student progression and the number of students they have.

In the article, we have shown how it is possible to discover demands from trace data left in the logs of information systems, which are used in accomplishing the organizations goals. When the use if IS in organizations is continuous and intensive, the data it generates becomes useful for analyses. Information about health-related or other outcomes may also be continuous and available in other information systems.

With the existence of these data sources, organizations possess the readiness to develop their operations and the well-being of their employees from their own starting points. Since Moodle is currently used in 242 different countries^22^, the opportunity to utilize the accumulated trace data with the help of our example is extensive. The example we have provided is likely to be useful also with trace data from other information systems and virtual platforms.

The existence of continuous data about job demands is a relevant issue also for researchers. The research on job burnout has shown that working under high or increasing (perceived) job demands for extended periods of time without the opportunity to recover is associated with an elevated risk of burnout^23^. Using trace data gathered from IS with outcome data on burnout could be an important addition to this field of research, enabling the objective and continuous measurement of job demands and controls as well as the analysis of their associations with burnout or employee wellbeing.

## Conclusion

In this article, we have shown how trace data can be used to analyze processes related to job demands using data-driven approaches. We have identified topics, themes, temporal processes, and employee clusters from Moodle data representing the work tasks of teachers. The information obtained is action-oriented, context-specific, and dynamic, meeting the current needs for information about the changing working life. The example we have provided could be widely utilized in organizations as well as in research on occupational wellbeing. At organizational level the approach could be used to identify periods of high job demands and launch interventions aimed at increasing employee wellbeing and reducing the risk of job burnout. For researchers the approach offers a novel method for the continuous measurement of job demands and controls that could be used to analyze the risk factors of job burnout. An important development would be the inclusion of additional data and prediction models on different outcomes such as sick leaves or employee turnover.

For applying our approach to different contexts and data, we suggest the following protocol:

1. Data gathering from the selected information system containing anonymized id, date of the recorded activity, and the performed action and target.
2. Data preprocessing, including the removal of target-action pairs that correspond the general use of the system instead of a specific activity by actors.
3. Defining the documents for LDA; accumulating words of target-action pairs over a week (or a time window of some other length) forms the bag of words for each actor for each week.
4. LDA-based topic modeling based on the weekly bag-of-words documents of all actors.
5. The assignment of a topic distribution for each actor for each recorded week.
6. The possible manual combination of the LDA topics into more general themes, i.e., the combination of the topic distributions into theme distributions by summing the probabilities of the topics in each theme. This step is optional and depends on the number of topics, and interpretability of the topics in the LDA model.
7. The computation of dissimilarities between all pairs of two actors. For this, a dissimilarity measure based on the weighted average of the Jensen-Shannon (JS) distances between the weekly theme vectors of the two actors has been used.
8. The clustering of the actors based on the dissimilarities between the actors. For this, complete-linkage agglomerative clustering has been applied.

## Data Availability

The datasets analyzed during the current study are not publicly available due to cooperation agreement. In the cooperation agreement with the organizations that provided the data, it has been agreed that the data is confidential and will not be disclosed to third parties. Data related questions should be addressed to T.K-L.

## Code availability

The data has been analyzed using commonly available models. In addition, an equation has been prepared for measuring the dissimilarity, which is available in the method section. All analyses were conducted using Python 3.10.
